# Supplementary material for: Cytotoxicity of Doxorubicin-Curcumin Nanoparticles Conjugated with Two Different Peptides (CKR and EVQ) against FLT3 Protein in Leukemic Stem Cells
Source: Polymers (Basel). 2024 Sep 2;16(17):2498. doi: 10.3390/polym16172498 (PMC11397985; doi:10.3390/polym16172498)
Supplement: Supplementary file 1 [file polymers-16-02498-s001.zip › polymers-3164205-supplementary.pdf]

Table S1. Particle size (PS), polydispersity index (PdI), and zeta potential (ZP) of various formulation of Dox-Cur-micelle (DCM) at day 0 and particle size at day 4 at room temperature (RT) (mean±SE, n=3)

**Formulation**	**Weight ratio of Dox: Cur (mg)**	**Weight ratio of Drugs: P407 (mg)**	**PS (nm) Day 0**	**PS (nm) Day 4**	**PdI Day 0**	**PdI Day 4**	**ZP (mV) Day 0**
DCM 1	1:9	1:20	27.40±2.28	29.82±4.35	0.162±0.034	0.178±0.041	−2.36±3.86
DCM 2	1:9	1:30	26.63±2.14	27.75±3.29	0.162±0.038	0.121±0.067	−3.64±4.45
DCM 3	1:9	1:40	25.41±1.52	26.16±2.18	0.164±0.030	0.164±0.034	−3.54±0.62
DCM 4	1:9	1:50	23.96±0.16	24.42±0.98	0.158±0.022	0.143±0.020	−4.27±4.95
DCM 5	1:9	1:60	22.90±0.63	23.25±0.15	0.148±0.030	0.176±0.017	−5.71±1.22
DCM 6	1:9	1:70	22.32±0.72	22.65±0.37	0.151±0.023	0.164±0.022	−5.83±0.81
DCM 7	1:9	1:80	21.50±0.97	21.58±0.68	0.150±0.018	0.154±0.020	−4.70±4.92

Table S2. Percentage of entrapment efficacy (%EE), and loading capacity (%LC) of various formulation of Dox-Cur-micelle (DCM) at day 0 at room temperature (RT) (mean±SE, n=3)

Formulation	Weight ratio of Dox: Cur (mg)	Weight ratio of Drugs: P407 (mg)	%EE of Dox Day 0	%LC of Dox Day 0	%EE of Cur Day 0	%LC of Cur Day 0
DCM 1	1:9	1:20	40.14±6.29	0.20±0.03	29.37±3.00	1.32±0.13
DCM 2	1:9	1:30	59.19±1.04	0.20±0.00	92.12±3.41	2.76±0.10
DCM 3	1:9	1:40	86.86±4.52	0.22±0.01	94.36±1.69	2.13±0.04
DCM 4	1:9	1:50	67.03±2.01	0.13±0.00	98.96±0.90	1.78±0.02
DCM 5	1:9	1:60	62.96±1.18	0.10±0.00	98.69±0.60	1.48±0.01
DCM 6	1:9	1:70	66.11±0.41	0.09±0.00	93.01±3.50	1.20±0.04
DCM 7	1:9	1:80	75.21±0.73	0.09±0.00	96.42±0.36	1.08±0.00

Table S3. Particle size (PS), polydispersity index (PdI), and zeta potential (ZP) of various formulation of Cur-micelle (CM) at day 0 and particle size and PdI at day 4 at RT (mean±SE, n=3)

Formulation	Weight ratio of Cur: P407 (mg)	PS (nm) Day 0	PS (nm) Day 4	PdI Day 0	PdI Day 4	ZP (mV) Day 0
**CM 1**	1:20	28.69±1.09	11509.67±89.12	0.179±0.009	0.418±0.020	–1.70±1.70
**CM 2**	1:24	25.53±1.06	8385.10±1195.00	0.092±0.003	0.197±0.018	–2.39±2.08
**CM 3**	1:30	24.99±0.95	4067.48±149.43	0.114±0.032	0.204±0.019	–1.68±1.32
**CM 4**	1:40	23.66±0.55	25.33±1.11	0.139±0.017	0.084±0.020	–2.88±1.63
**CM 5**	1:50	25.31±1.26	26.30±2.60	0.098±0.004	0.088±0.014	–2.00±1.93
**CM 6**	1:60	23.68±0.16	24.62±0.71	0.118±0.006	0.103±0.017	–3.51±0.28
**CM 7**	1:80	25.54±1.47	25.86±1.62	0.147±0.008	0.127±0.020	–3.06±1.50

Table S4. Percentage of entrapment efficacy (%EE), and loading capacity (%LC) of various formulation of Cur-micelle (CM) at day 0 and particle size and PdI at day 4 at RT (mean±SE, n=3)

Formulation	Weight ratio of Cur: P407 (mg)	%EE of Cur Day 0	%LC of Cur Day 0
**CM 1**	1:20	24.58±1.22	1.23±0.06
**CM 2**	1:24	73.16±3.60	3.05±0.15
**CM 3**	1:30	83.46±4.10	2.78±0.14
**CM 4**	1:40	83.81±4.03	2.10±0.10
**CM 5**	1:50	75.67±2.70	1.86±0.38
**CM 6**	1:60	76.02±2.64	1.49±0.25
**CM 7**	1:80	81.57±2.74	1.61±0.61

Table S5. Particle size, polydispersity index (PdI), zeta potential (ZP), percentage of entrapment efficacy (%EE), and loading capacity (%LC) of DCM conjugated with or without peptides at day 0, RT (mean±SE, n=3).

Formulation	Size (nm)	Pd	ZP (mV)	%EE of Dox	%LC of Dox	%EE of Cur	%LC of Cur	Average weight ratio of Dox: Cur
**DCM**	25.04±0.28	0.183±0.028	–3.54±0.62	86.86±4.52	0.22±0.01	94.36±1.69	2.13±0.04	1:9.9
**DCM-C**	39.90±4.46	0.245±0.022	–4.22±2.59	94.20±3.93	0.24±0.01	72.38±9.78	1.63±0.22	1:6.9
**DCM-E**	37.29±4.14	0.223±0.042	–4.83±5.09	90.08±4.75	0.23±0.01	86.91±5.71	1.95±0.13	1:8.7
**DCM-C+E**	37.36±5.54	0.241±0.032	–5.92±2.00	96.10±3.86	0.24±0.01	79.37±7.50	1.79±0.17	1:7.5

Table S6. Particle size, polydispersity index (PdI), zeta potential (ZP), percentage of entrapment efficacy (%EE), and loading capacity (%LC) of CM conjugated with or without peptides at day 0, RT (mean±SE, n=3).

Formulation	Size (nm)	PdI	ZP (mV)	%EE of Cur	%LC of Cur
**CM**	23.64±0.24	0.108±0.013	–0.63±0.15	91.01±4.24	1.88±0.35
**CM-C**	29.08±0.25	0.145±0.018	–2.23±0.99	71.14±5.74	1.78±0.14
**CM-E**	45.29±9.93	0.290±0.099	–0.83±0.30	80.06±9.52	1.83±0.40
**CM-C+E**	36.68±5.01	0.251±0.087	–2.23±0.98	86.28±7.72	1.95±0.38
